# Diaqua­[μ-11,23-di-*tert*-butyl-3,7,15,19-tetra­azatricyclo­[19.3.1.1^9,13^]tetra­cosa-1(25),2,6,9,11,13(26),14,19,21,23-do­decaene-25,26-diolato-κ^4^ *N* ^3^,*N* ^7^,*O* ^25^,*O* ^26^:κ^4^ *N* ^15^,*N* ^19^,*O* ^25^,*O* ^26^]dicopper(II) bis­(perchlorate)

**DOI:** 10.1107/S1600536812031248

**Published:** 2012-07-14

**Authors:** Qiang Xu, Zhaodong Wang, Jiahong He, Zhongrong Song, Fengming Chen, Jiangping Meng, Chengbo Hu

**Affiliations:** a School of Materials and Chemical Engineering, Chongqing University of Arts and Sciences, Yongchuan, Chongqing 402160, People’s Republic of China

## Abstract

In the dinuclear title complex, [Cu_2_(C_30_H_38_N_4_O_2_)(H_2_O)_2_](ClO_4_)_2_, the coordination cation has crystallographically imposed twofold rotational symmetry. The Cu^II^ ion is five-coordinated by two N and two O atoms from the macrocylic ligand and one O atom from a water mol­ecule, forming a square-pyramidal N_2_O_3_ geometry with the water mol­ecule in the apical position. The distance between the two Cu^II^ atoms is 3.0930 (5) Å. Hydrogen bonds between water mol­ecules and between water mol­ecules and perchlorate anions assemble two cations and four anions into discrete supermolecules of *S*_4_ symmetry. Intramolecular O—H⋯N hydrogen bonds are also observed. The perchlorate anion and the *tert*-butyl group are disordered over two positions, with occupancies of the major positions of 0.527 (11) and 0.592 (9), respectively.

## Related literature

For the synthesis of the magnesium precursor, see: Mohanta *et al. *(1997 ▶). For the synthesis of 4-*tert*-butyl-2,6-diformyl­phenol, see: Lindoy *et al.* (1998 ▶). For similar copper(II) and nickel(II) complexes, see: Bai *et al.* (2007 ▶); Chen *et al.* (2005 ▶); Nanda *et al.* (1994 ▶). For the preparation of similar macrocyclic ligands, see: Thompson *et al.* (1996 ▶); Pilkington & Robson (1970 ▶); Zhou *et al.* (2005 ▶).
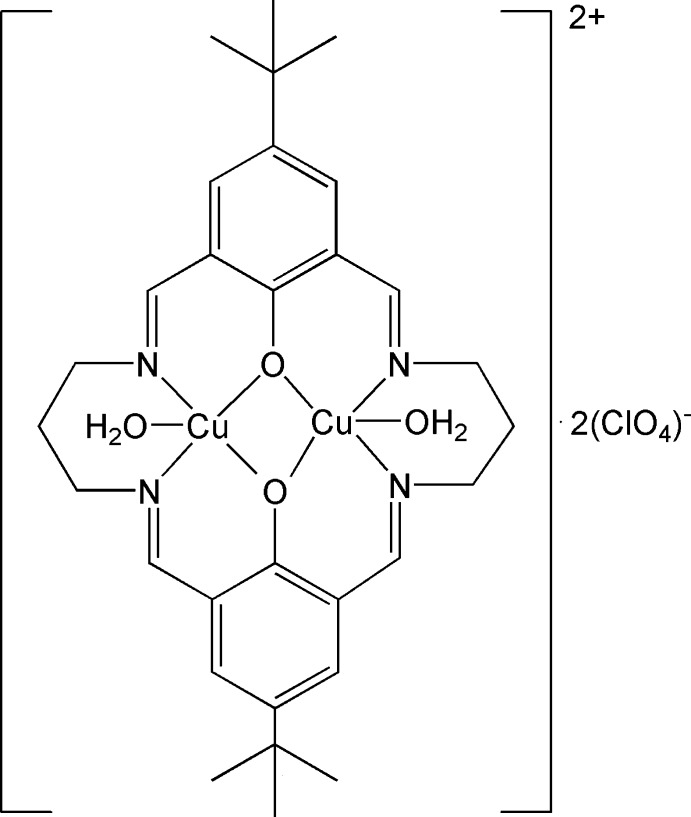


## Experimental

### 

#### Crystal data

[Cu_2_(C_30_H_38_N_4_O_2_)(H_2_O)_2_](ClO_4_)_2_
*M* *_r_* = 848.66
Tetragonal, 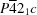

*a* = 18.9013 (4) Å
*c* = 9.9174 (4) Å
*V* = 3543.08 (18) Å^3^
*Z* = 4
Mo *K*α radiation
μ = 1.42 mm^−1^
*T* = 296 K
0.38 × 0.36 × 0.32 mm

#### Data collection

Bruker APEXII CCD diffractometer
Absorption correction: multi-scan (*SADABS*; Bruker, 2008 ▶) *T*_min_ = 0.615, *T*_max_ = 0.660
19006 measured reflections
3489 independent reflections
2942 reflections with *I* > 2σ(*I*)
*R*_int_ = 0.021

#### Refinement

*R*[*F* ^2^ > 2σ(*F* ^2^)] = 0.039
*wR*(*F* ^2^) = 0.115
*S* = 1.02
3489 reflections
298 parameters
125 restraints
H atoms treated by a mixture of independent and constrained refinement
Δρ_max_ = 0.47 e Å^−3^
Δρ_min_ = −0.33 e Å^−3^
Absolute structure: Flack (1983 ▶), 1527 Friedel pairs
Flack parameter: 0.31 (3)

### 

Data collection: *APEX2* (Bruker, 2008 ▶); cell refinement: *SAINT* (Bruker, 2008 ▶); data reduction: *SAINT*; program(s) used to solve structure: *SHELXS97* (Sheldrick, 2008 ▶); program(s) used to refine structure: *SHELXL97* (Sheldrick, 2008 ▶); molecular graphics: *SHELXTL* (Sheldrick, 2008 ▶); software used to prepare material for publication: *SHELXTL*.

## Supplementary Material

Crystal structure: contains datablock(s) I, global. DOI: 10.1107/S1600536812031248/gk2504sup1.cif

Structure factors: contains datablock(s) I. DOI: 10.1107/S1600536812031248/gk2504Isup2.hkl

Additional supplementary materials:  crystallographic information; 3D view; checkCIF report

## Figures and Tables

**Table 1:** Selected bond lengths (Å)

Cu1—N1	1.939 (4)
Cu1—N2	1.945 (4)
Cu1—O1^i^	1.954 (3)
Cu1—O1	1.964 (3)
Cu1—O2	2.707 (5)

Symmetry code: (i) 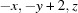.

**Table 2:** Hydrogen-bond geometry (Å, °)

*D*—H⋯*A*	*D*—H	H⋯*A*	*D*⋯*A*	*D*—H⋯*A*
O2—H2*B*⋯O2^ii^	0.82 (2)	2.07 (4)	2.821 (6)	153 (8)
O2—H2*A*⋯O3	0.81 (2)	2.26 (3)	2.806 (8)	125 (2)
O2—H2*A*⋯O3′	0.81 (2)	2.49 (4)	2.947 (10)	117 (3)
O2—H2*A*⋯N1	0.81 (2)	2.55 (3)	3.197 (6)	138 (3)

Symmetry code: (ii) 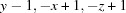.

**Fig. 1.**
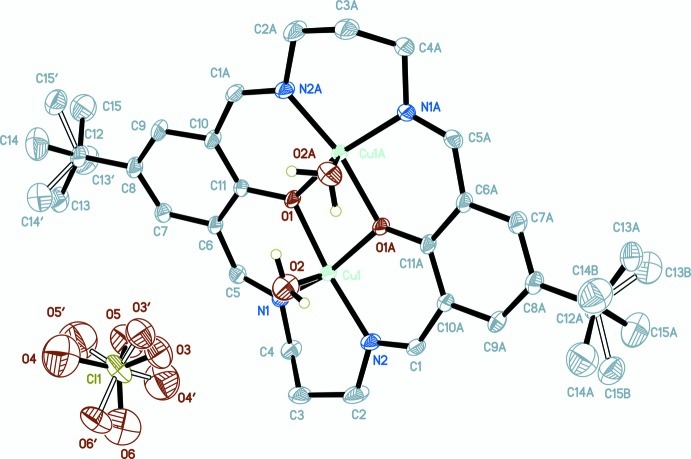
Molecular structure of the title compound with displacement ellipsoids drawn at the 30% probability level. The H atoms attached to C atoms were omitted for clarity. Atoms with the A label are generated by the '-x, -y+2, z' symmetry operation. tert-Butyl group and perchlorate anion are disordered.

**Fig. 2.**
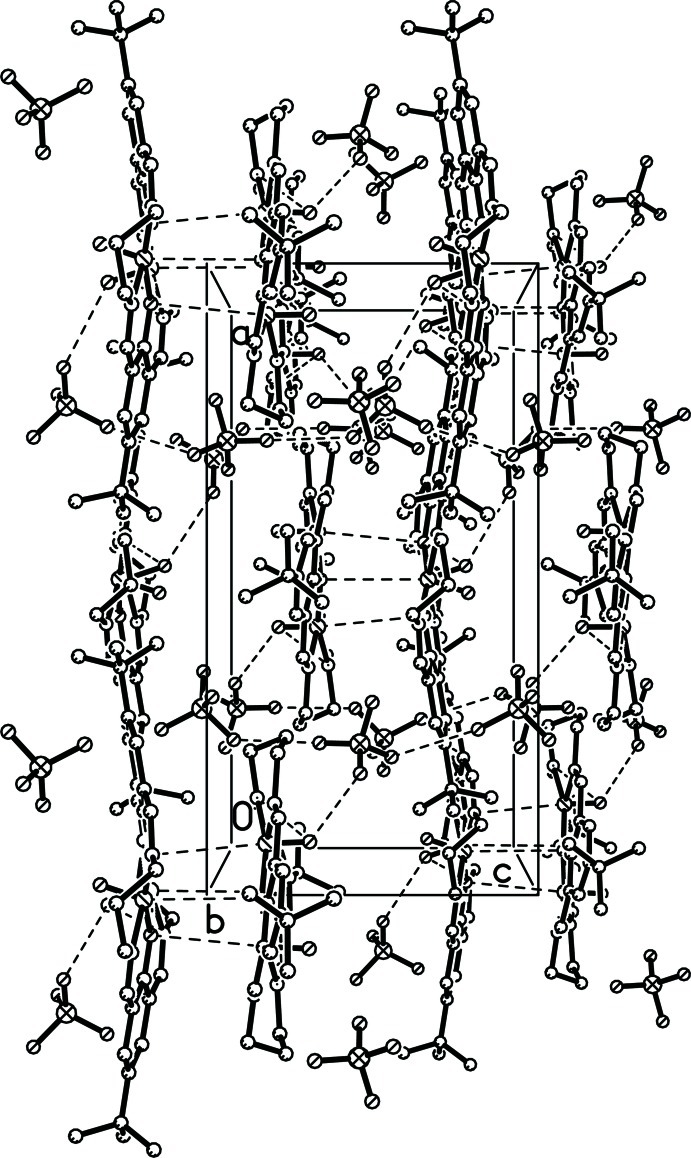
View of the crystal packing along the b axis. For the sake of clarity, H atoms and minor position of the disordered groups have been omitted.

## supplementary crystallographic information

### Comment

Dinuclear heterometallic and homometallic transition metal complexes have been well studied with a series of macrocyclic liagnds based on the first reported condensation reaction between 2,6-diformyl-4-R-phenol (*R*= CH_3_, Cl, F, *n*-butyl) and alkylenediamine by stepwise template reaction (Thompson *et al.*, 1996; Pilkington & Robson, 1970; Zhou *et al.*, 2005). Several tetranuclear as well as trinuclear nickel(II) and copper(II) complexes have been structurally characterized (Mohanta *et al.*,1997; Nanda *et al.*,1994). In addition, Mohanta *et al.* (1997) reported a protonated macrocyclic magnesium compound of composition [Mg_2_(*L*^1^H_4_)_2_(NO_3_)_2_](NO_3_)_2_6H_2_O by a template reaction. The transmetalation reaction of the magnesium precursor with copper(II) perchlorate in the presence of triethylamine resulted in the formation of a dinuclear copper(II) complex (Mohanta *et al.*,1997). Herein, we synthesized a similar magnesium precursor by a template reaction involving 4-*tert*-butyl-2,6-diformylphenol, 1,3-diaminopropane, magnesium acetate, and magnesium nitrate.The transmetalation reaction of the new magnesium precursor with copper(II) perchlorate leads to a new dinuclear copper(II) complex.

The structure of the cation the title compound is shown in Fig.1. In the cation, each copper(II) is coordinated by two O atoms and two N atoms from the macrocylic ligand and one O from water molecule, forming a square pyramidal {N_2_O_3_} geometry. In {N_2_O_3_}, the N_2_O_2_ donor sets from the macrocyclic ligand occupy the basal plane of the pyramid and the O atom from the water molecule locates in the apical position. The distance of the apical O atom and the copper atom [Cu1–O2: 2.707 (5) Å] is longer than the basal donors [ranging from 1.938 (4) to 1.964 (3) Å] due to the Jahn-Teller effect. Fig. 2 shows the crystal packing of the title compound along the *b* axis.

### Experimental

4-*tert*-Butyl-2,6-diformylphenol was synthesized according to the reported literature (Lindoy *et al.*,1998). The magnesium precursor was prepared according to the reported method by Mohanta (Mohanta *et al.*,1997). The title complex was obtained by the following procedures: Cu(ClO_4_)_2_6H_2_O (0.185 g, 0.5 mmol) and magnesium precursor (0.136 g, 0.1 mmol) were dissolved in CH_3_OH (15 ml) and the solution was filtered and left to stand at room temperature. Blue block single crystals suitable for X-ray analysis were obtained by slow evaporation over a period of two weeks.

### Refinement

The H atoms bonded to C atoms were positioned geometrically and refined using a riding model, with C–H = 0.93-0.96 Å, and with *U*_iso_(H) = 1.2 or 1.5 times *U*_eq_(C). The H atoms bonded to O1W were located in Fourier difference maps and refined with restraints imposed on O-H and H···H distances [O-H= 0.83 (1) Å, H···H. 1.35 (1) Å]. Restraints were also imposed on Cl-O, O···O, C-C and C···C distances of disordered perchlorate and tert-butyl groups [Cl-O 1.44 (1) Å, O···O 2.35 (1) Å, C-C 1. 50 (1) Å, C···C 2.35 (1) Å]. The crystal was refined as an inversion twin with the ratio of the two twin domains of 0.31 (3):0.69 (3).

### Crystal data

**Table:**

[Cu_2_(C_30_H_38_N_4_O_2_)(H_2_O)_2_](ClO_4_)_2_	*D*_x_ = 1.591 Mg m^−^^3^
*M**_r_* = 848.66	Mo *K*α radiation, λ = 0.71073 Å
Tetragonal, *P*42_1_*c*	Cell parameters from 7958 reflections
Hall symbol: P-42 n	θ = 2.3–29.4°
*a* = 18.9013 (4) Å	µ = 1.42 mm^−^^1^
*c* = 9.9174 (4) Å	*T* = 296 K
*V* = 3543.08 (18) Å^3^	Block, blue
*Z* = 4	0.38 × 0.36 × 0.32 mm
*F*(000) = 1752	

### Data collection

**Table:**

Bruker APEXII CCD diffractometer	3489 independent reflections
Radiation source: fine-focus sealed tube	2942 reflections with *I* > 2σ(*I*)
Graphite monochromator	*R*_int_ = 0.021
phi and ω scans	θ_max_ = 26.0°, θ_min_ = 1.5°
Absorption correction: multi-scan (*SADABS*; Bruker, 2008)	*h* = −18→23
*T*_min_ = 0.615, *T*_max_ = 0.660	*k* = −22→23
19006 measured reflections	*l* = −12→9

### Refinement

**Table:**

Refinement on *F*^2^	Secondary atom site location: difference Fourier map
Least-squares matrix: full	Hydrogen site location: inferred from neighbouring sites
*R*[*F*^2^ > 2σ(*F*^2^)] = 0.039	H atoms treated by a mixture of independent and constrained refinement
*wR*(*F*^2^) = 0.115	*w* = 1/[σ^2^(*F*_o_^2^) + (0.0615*P*)^2^ + 4.6836*P*] where *P* = (*F*_o_^2^ + 2*F*_c_^2^)/3
*S* = 1.02	(Δ/σ)_max_ = 0.001
3489 reflections	Δρ_max_ = 0.47 e Å^−^^3^
298 parameters	Δρ_min_ = −0.33 e Å^−^^3^
125 restraints	Absolute structure: Flack (1983), 1527 Friedel pairs
Primary atom site location: structure-invariant direct methods	Flack parameter: 0.31 (3)

### Special details

**Table:**

Geometry. All e.s.d.'s (except the e.s.d. in the dihedral angle between two l.s. planes) are estimated using the full covariance matrix. The cell e.s.d.'s are taken into account individually in the estimation of e.s.d.'s in distances, angles and torsion angles; correlations between e.s.d.'s in cell parameters are only used when they are defined by crystal symmetry. An approximate (isotropic) treatment of cell e.s.d.'s is used for estimating e.s.d.'s involving l.s. planes.

### Fractional atomic coordinates and isotropic or equivalent isotropic displacement parameters (Å^2^)

**Table:**

	*x*	*y*	*z*	*U*_iso_*/*U*_eq_	Occ. (<1)
Cu1	0.08182 (2)	0.99986 (3)	0.18030 (5)	0.03063 (16)	
O1	−0.00045 (19)	0.93667 (12)	0.1690 (3)	0.0329 (6)	
O2	0.0833 (2)	0.9539 (2)	0.4388 (5)	0.0614 (10)	
N1	0.14955 (18)	0.92478 (19)	0.1474 (4)	0.0314 (9)	
N2	0.14970 (19)	1.0758 (2)	0.2082 (4)	0.0389 (10)	
C1	0.1312 (2)	1.1411 (2)	0.2233 (5)	0.0361 (10)	
H1	0.1678	1.1728	0.2406	0.043*	
C2	0.2279 (3)	1.0650 (3)	0.2171 (8)	0.0657 (19)	
H2A'	0.2493	1.0819	0.1343	0.079*	
H2B'	0.2461	1.0936	0.2904	0.079*	
C3	0.2500 (2)	0.9908 (3)	0.2390 (6)	0.0504 (13)	
H3A	0.2311	0.9744	0.3244	0.061*	
H3B	0.3012	0.9889	0.2446	0.061*	
C4	0.2254 (2)	0.9415 (3)	0.1278 (5)	0.0431 (11)	
H4A	0.2322	0.9639	0.0408	0.052*	
H4B	0.2530	0.8983	0.1296	0.052*	
C5	0.1332 (2)	0.8591 (2)	0.1536 (5)	0.0347 (10)	
H5	0.1698	0.8272	0.1384	0.042*	
C6	0.0651 (2)	0.8288 (2)	0.1813 (5)	0.0318 (9)	
C7	0.0649 (2)	0.7551 (2)	0.2042 (5)	0.0398 (11)	
H7	0.1073	0.7305	0.1974	0.048*	
C8	0.0046 (3)	0.71818 (19)	0.2360 (4)	0.0393 (9)	
C9	−0.0578 (3)	0.7557 (2)	0.2404 (5)	0.0394 (11)	
H9	−0.0995	0.7316	0.2599	0.047*	
C10	−0.0611 (3)	0.8288 (2)	0.2168 (5)	0.0330 (11)	
C11	0.0005 (3)	0.86673 (17)	0.1894 (4)	0.0297 (7)	
C12	0.0062 (3)	0.6385 (2)	0.2598 (5)	0.0538 (12)	
C13	0.0775 (4)	0.6074 (4)	0.2618 (11)	0.059 (3)	0.592 (9)
H13A	0.0741	0.5574	0.2771	0.089*	0.592 (9)
H13B	0.1047	0.6287	0.3328	0.089*	0.592 (9)
H13C	0.1004	0.6159	0.1768	0.089*	0.592 (9)
C14	−0.0277 (6)	0.6227 (6)	0.3911 (9)	0.085 (4)	0.592 (9)
H14A	−0.0269	0.5725	0.4067	0.127*	0.592 (9)
H14B	−0.0759	0.6389	0.3898	0.127*	0.592 (9)
H14C	−0.0024	0.6463	0.4619	0.127*	0.592 (9)
C15	−0.0368 (6)	0.6024 (6)	0.1554 (11)	0.086 (4)	0.592 (9)
H15A	−0.0357	0.5522	0.1705	0.129*	0.592 (9)
H15B	−0.0178	0.6127	0.0678	0.129*	0.592 (9)
H15C	−0.0848	0.6188	0.1603	0.129*	0.592 (9)
C13'	0.0409 (8)	0.6032 (9)	0.1429 (13)	0.084 (6)	0.408 (9)
H13D	0.0419	0.5530	0.1577	0.126*	0.408 (9)
H13E	0.0883	0.6206	0.1335	0.126*	0.408 (9)
H13F	0.0147	0.6132	0.0622	0.126*	0.408 (9)
C14'	0.0491 (8)	0.6226 (10)	0.3793 (13)	0.085 (6)	0.408 (9)
H14D	0.0500	0.5724	0.3938	0.127*	0.408 (9)
H14E	0.0289	0.6455	0.4567	0.127*	0.408 (9)
H14F	0.0964	0.6395	0.3657	0.127*	0.408 (9)
C15'	−0.0633 (5)	0.6048 (7)	0.2754 (15)	0.064 (4)	0.408 (9)
H15D	−0.0572	0.5550	0.2896	0.096*	0.408 (9)
H15E	−0.0908	0.6124	0.1953	0.096*	0.408 (9)
H15F	−0.0873	0.6251	0.3514	0.096*	0.408 (9)
Cl1	0.22950 (8)	0.78567 (9)	0.46056 (14)	0.0669 (4)	
O3	0.1844 (6)	0.8458 (5)	0.4620 (12)	0.086 (4)	0.527 (11)
O4	0.2055 (8)	0.7328 (7)	0.5596 (13)	0.160 (7)	0.527 (11)
O5	0.2295 (6)	0.7511 (6)	0.3334 (8)	0.102 (4)	0.527 (11)
O6	0.2988 (5)	0.8071 (8)	0.4988 (16)	0.153 (6)	0.527 (11)
O3'	0.1632 (4)	0.8212 (7)	0.4726 (13)	0.082 (4)	0.473 (11)
O4'	0.2672 (7)	0.8119 (8)	0.3419 (11)	0.142 (7)	0.473 (11)
O5'	0.2189 (8)	0.7114 (4)	0.4411 (19)	0.164 (7)	0.473 (11)
O6'	0.2725 (5)	0.7960 (6)	0.5766 (10)	0.084 (4)	0.473 (11)
H2B	0.054 (4)	0.932 (4)	0.482 (8)	0.101*	
H2A	0.115 (3)	0.937 (3)	0.395 (3)	0.101*	

### Atomic displacement parameters (Å^2^)

**Table:**

	*U*^11^	*U*^22^	*U*^33^	*U*^12^	*U*^13^	*U*^23^
Cu1	0.0211 (2)	0.0202 (2)	0.0506 (3)	0.0005 (3)	−0.00050 (19)	−0.0018 (3)
O1	0.0256 (12)	0.0176 (11)	0.0556 (16)	0.0010 (15)	0.002 (2)	0.0020 (10)
O2	0.051 (2)	0.056 (2)	0.078 (3)	0.0051 (18)	0.013 (2)	−0.008 (2)
N1	0.0249 (17)	0.0275 (18)	0.042 (2)	0.0022 (15)	0.0023 (15)	−0.0005 (15)
N2	0.0228 (17)	0.031 (2)	0.063 (3)	−0.0002 (15)	−0.0023 (17)	−0.0026 (18)
C1	0.030 (2)	0.027 (2)	0.052 (3)	−0.0061 (17)	−0.004 (2)	−0.0028 (19)
C2	0.026 (2)	0.037 (3)	0.134 (6)	0.001 (2)	−0.013 (3)	−0.016 (3)
C3	0.027 (2)	0.049 (3)	0.076 (3)	0.002 (2)	−0.009 (2)	−0.003 (3)
C4	0.030 (2)	0.033 (2)	0.066 (3)	0.0016 (19)	0.006 (2)	−0.006 (2)
C5	0.029 (2)	0.029 (2)	0.046 (3)	0.0074 (18)	0.0041 (19)	0.0023 (19)
C6	0.034 (2)	0.019 (2)	0.043 (2)	0.0015 (17)	−0.001 (2)	0.0013 (19)
C7	0.036 (2)	0.024 (2)	0.060 (3)	0.0062 (19)	−0.002 (2)	0.003 (2)
C8	0.046 (2)	0.0224 (17)	0.049 (2)	0.001 (2)	0.000 (2)	0.0035 (16)
C9	0.047 (3)	0.022 (2)	0.049 (3)	−0.006 (2)	0.004 (2)	0.0032 (19)
C10	0.034 (3)	0.022 (2)	0.043 (3)	−0.0023 (17)	0.0053 (19)	0.0008 (17)
C11	0.0305 (17)	0.0183 (15)	0.040 (2)	0.002 (2)	−0.004 (2)	0.0004 (14)
C12	0.054 (3)	0.0230 (18)	0.085 (3)	0.001 (2)	−0.005 (3)	0.010 (2)
C13	0.062 (4)	0.031 (4)	0.085 (5)	0.013 (3)	0.003 (4)	0.010 (3)
C14	0.097 (6)	0.066 (5)	0.091 (6)	0.013 (4)	0.012 (4)	0.020 (4)
C15	0.095 (6)	0.061 (5)	0.102 (6)	−0.006 (4)	−0.015 (5)	−0.001 (4)
C13'	0.092 (8)	0.068 (7)	0.092 (8)	−0.002 (5)	0.011 (5)	−0.008 (5)
C14'	0.088 (7)	0.075 (7)	0.091 (7)	−0.001 (5)	−0.008 (5)	0.015 (5)
C15'	0.070 (6)	0.048 (6)	0.075 (6)	−0.007 (4)	0.002 (5)	0.007 (4)
Cl1	0.0723 (9)	0.0795 (10)	0.0488 (7)	0.0325 (8)	−0.0097 (7)	−0.0036 (7)
O3	0.088 (5)	0.075 (5)	0.095 (5)	0.031 (4)	−0.004 (4)	0.003 (4)
O4	0.169 (8)	0.154 (8)	0.157 (8)	0.000 (5)	0.014 (5)	0.012 (5)
O5	0.105 (6)	0.110 (6)	0.091 (5)	0.033 (4)	0.002 (4)	−0.021 (4)
O6	0.142 (8)	0.159 (8)	0.157 (8)	−0.004 (5)	−0.012 (5)	0.004 (5)
O3'	0.076 (6)	0.082 (6)	0.087 (6)	0.019 (4)	0.002 (4)	−0.001 (4)
O4'	0.137 (8)	0.151 (8)	0.137 (8)	0.011 (5)	0.013 (5)	0.013 (5)
O5'	0.169 (9)	0.151 (8)	0.173 (9)	−0.002 (5)	0.002 (5)	−0.011 (5)
O6'	0.072 (5)	0.098 (6)	0.082 (5)	0.022 (4)	−0.026 (4)	−0.008 (4)

### Geometric parameters (Å, º)

**Table:**

Cu1—N1	1.939 (4)	C10—C1^i^	1.444 (6)
Cu1—N2	1.945 (4)	C12—C15'	1.467 (8)
Cu1—O1^i^	1.954 (3)	C12—C14'	1.468 (8)
Cu1—O1	1.964 (3)	C12—C13	1.471 (7)
Cu1—O2	2.707 (5)	C12—C14	1.482 (7)
O1—C11	1.338 (4)	C12—C15	1.482 (8)
O1—Cu1^i^	1.954 (3)	C12—C13'	1.489 (9)
O2—H2B	0.82 (2)	C13—H13A	0.9600
O2—H2A	0.81 (2)	C13—H13B	0.9600
N1—C5	1.282 (6)	C13—H13C	0.9600
N1—C4	1.481 (6)	C14—H14A	0.9600
N2—C1	1.291 (6)	C14—H14B	0.9600
N2—C2	1.494 (6)	C14—H14C	0.9600
C1—C10^i^	1.444 (6)	C15—H15A	0.9600
C1—H1	0.9300	C15—H15B	0.9600
C2—C3	1.480 (7)	C15—H15C	0.9600
C2—H2A'	0.9700	C13'—H13D	0.9600
C2—H2B'	0.9700	C13'—H13E	0.9600
C3—C4	1.516 (7)	C13'—H13F	0.9600
C3—H3A	0.9700	C14'—H14D	0.9600
C3—H3B	0.9700	C14'—H14E	0.9600
C4—H4A	0.9700	C14'—H14F	0.9600
C4—H4B	0.9700	C15'—H15D	0.9600
C5—C6	1.435 (6)	C15'—H15E	0.9600
C5—H5	0.9300	C15'—H15F	0.9600
C6—C7	1.411 (6)	Cl1—O5	1.421 (6)
C6—C11	1.418 (6)	Cl1—O3	1.421 (6)
C7—C8	1.372 (7)	Cl1—O6'	1.422 (7)
C7—H7	0.9300	Cl1—O6	1.422 (7)
C8—C9	1.378 (7)	Cl1—O3'	1.426 (7)
C8—C12	1.525 (5)	Cl1—O5'	1.431 (7)
C9—C10	1.403 (6)	Cl1—O4'	1.462 (8)
C9—H9	0.9300	Cl1—O4	1.472 (8)
C10—C11	1.395 (7)		
			
N1—Cu1—N2	97.38 (14)	C13—C12—C14	107.7 (6)
N1—Cu1—O1^i^	163.64 (15)	C13—C12—C15	109.2 (6)
N2—Cu1—O1^i^	94.27 (13)	C14—C12—C15	106.5 (6)
N1—Cu1—O1	93.91 (13)	C15'—C12—C13'	106.4 (7)
N2—Cu1—O1	168.30 (15)	C14'—C12—C13'	107.1 (7)
O1^i^—Cu1—O1	75.33 (11)	C15'—C12—C8	115.3 (7)
C11—O1—Cu1^i^	127.5 (3)	C14'—C12—C8	109.8 (8)
C11—O1—Cu1	125.6 (3)	C13—C12—C8	114.4 (5)
Cu1^i^—O1—Cu1	104.28 (11)	C14—C12—C8	109.1 (6)
H2B—O2—H2A	125 (8)	C15—C12—C8	109.7 (6)
C5—N1—C4	116.5 (4)	C13'—C12—C8	109.3 (8)
C5—N1—Cu1	122.8 (3)	C12—C13—H13A	109.5
C4—N1—Cu1	120.3 (3)	C12—C13—H13B	109.5
C1—N2—C2	113.0 (4)	H13A—C13—H13B	109.5
C1—N2—Cu1	122.9 (3)	C12—C13—H13C	109.5
C2—N2—Cu1	124.1 (3)	H13A—C13—H13C	109.5
N2—C1—C10^i^	128.3 (4)	H13B—C13—H13C	109.5
N2—C1—H1	115.9	C12—C14—H14A	109.5
C10^i^—C1—H1	115.9	C12—C14—H14B	109.5
C3—C2—N2	114.7 (4)	H14A—C14—H14B	109.5
C3—C2—H2A'	108.6	C12—C14—H14C	109.5
N2—C2—H2A'	108.6	H14A—C14—H14C	109.5
C3—C2—H2B'	108.6	H14B—C14—H14C	109.5
N2—C2—H2B'	108.6	C12—C15—H15A	109.5
H2A'—C2—H2B'	107.6	C12—C15—H15B	109.5
C2—C3—C4	112.8 (5)	H15A—C15—H15B	109.5
C2—C3—H3A	109.0	C12—C15—H15C	109.5
C4—C3—H3A	109.0	H15A—C15—H15C	109.5
C2—C3—H3B	109.0	H15B—C15—H15C	109.5
C4—C3—H3B	109.0	C12—C13'—H13D	109.5
H3A—C3—H3B	107.8	C12—C13'—H13E	109.5
N1—C4—C3	109.5 (4)	H13D—C13'—H13E	109.5
N1—C4—H4A	109.8	C12—C13'—H13F	109.5
C3—C4—H4A	109.8	H13D—C13'—H13F	109.5
N1—C4—H4B	109.8	H13E—C13'—H13F	109.5
C3—C4—H4B	109.8	C12—C14'—H14D	109.5
H4A—C4—H4B	108.2	C12—C14'—H14E	109.5
N1—C5—C6	127.7 (4)	H14D—C14'—H14E	109.5
N1—C5—H5	116.1	C12—C14'—H14F	109.5
C6—C5—H5	116.1	H14D—C14'—H14F	109.5
C7—C6—C11	119.2 (4)	H14E—C14'—H14F	109.5
C7—C6—C5	115.3 (4)	C12—C15'—H15D	109.5
C11—C6—C5	125.6 (4)	C12—C15'—H15E	109.5
C8—C7—C6	122.8 (4)	H15D—C15'—H15E	109.5
C8—C7—H7	118.6	C12—C15'—H15F	109.5
C6—C7—H7	118.6	H15D—C15'—H15F	109.5
C7—C8—C9	117.2 (3)	H15E—C15'—H15F	109.5
C7—C8—C12	121.5 (5)	O5—Cl1—O3	112.1 (6)
C9—C8—C12	121.3 (5)	O5—Cl1—O6	111.6 (6)
C8—C9—C10	122.6 (4)	O3—Cl1—O6	108.8 (7)
C8—C9—H9	118.7	O6'—Cl1—O3'	111.6 (6)
C10—C9—H9	118.7	O6'—Cl1—O5'	108.9 (7)
C11—C10—C9	120.2 (4)	O3'—Cl1—O5'	110.5 (6)
C11—C10—C1^i^	124.9 (4)	O6'—Cl1—O4'	109.0 (6)
C9—C10—C1^i^	114.9 (4)	O3'—Cl1—O4'	109.7 (6)
O1—C11—C10	121.7 (4)	O5'—Cl1—O4'	107.0 (7)
O1—C11—C6	120.2 (4)	O5—Cl1—O4	106.3 (6)
C10—C11—C6	118.0 (3)	O3—Cl1—O4	110.5 (6)
C15'—C12—C14'	108.7 (7)	O6—Cl1—O4	107.4 (6)
			
N1—Cu1—O1—C11	23.1 (3)	C5—C6—C7—C8	178.0 (5)
N2—Cu1—O1—C11	−141.7 (7)	C6—C7—C8—C9	2.3 (7)
O1^i^—Cu1—O1—C11	−169.4 (3)	C6—C7—C8—C12	179.9 (5)
N1—Cu1—O1—Cu1^i^	−174.27 (14)	C7—C8—C9—C10	−1.4 (7)
N2—Cu1—O1—Cu1^i^	21.0 (8)	C12—C8—C9—C10	−178.9 (5)
O1^i^—Cu1—O1—Cu1^i^	−6.78 (17)	C8—C9—C10—C11	−0.9 (8)
N2—Cu1—N1—C5	163.2 (4)	C8—C9—C10—C1^i^	178.9 (4)
O1^i^—Cu1—N1—C5	−61.8 (7)	Cu1^i^—O1—C11—C10	3.0 (5)
O1—Cu1—N1—C5	−13.7 (4)	Cu1—O1—C11—C10	161.7 (3)
N2—Cu1—N1—C4	−10.2 (4)	Cu1^i^—O1—C11—C6	−177.9 (3)
O1^i^—Cu1—N1—C4	124.9 (5)	Cu1—O1—C11—C6	−19.3 (5)
O1—Cu1—N1—C4	172.9 (3)	C9—C10—C11—O1	−178.7 (4)
N1—Cu1—N2—C1	173.9 (4)	C1^i^—C10—C11—O1	1.5 (7)
O1^i^—Cu1—N2—C1	5.4 (4)	C9—C10—C11—C6	2.3 (6)
O1—Cu1—N2—C1	−21.5 (10)	C1^i^—C10—C11—C6	−177.5 (5)
N1—Cu1—N2—C2	−7.4 (5)	C7—C6—C11—O1	179.5 (4)
O1^i^—Cu1—N2—C2	−175.8 (5)	C5—C6—C11—O1	0.7 (7)
O1—Cu1—N2—C2	157.3 (7)	C7—C6—C11—C10	−1.4 (7)
C2—N2—C1—C10^i^	178.0 (5)	C5—C6—C11—C10	179.8 (5)
Cu1—N2—C1—C10^i^	−3.2 (7)	C7—C8—C12—C15'	−172.3 (8)
C1—N2—C2—C3	163.5 (5)	C9—C8—C12—C15'	5.1 (9)
Cu1—N2—C2—C3	−15.3 (8)	C7—C8—C12—C14'	64.5 (9)
N2—C2—C3—C4	59.5 (7)	C9—C8—C12—C14'	−118.0 (8)
C5—N1—C4—C3	−125.1 (4)	C7—C8—C12—C13	7.0 (8)
Cu1—N1—C4—C3	48.7 (5)	C9—C8—C12—C13	−175.5 (6)
C2—C3—C4—N1	−77.9 (6)	C7—C8—C12—C14	127.7 (7)
C4—N1—C5—C6	175.2 (5)	C9—C8—C12—C14	−54.8 (7)
Cu1—N1—C5—C6	1.6 (7)	C7—C8—C12—C15	−116.1 (7)
N1—C5—C6—C7	−169.8 (5)	C9—C8—C12—C15	61.4 (8)
N1—C5—C6—C11	9.0 (9)	C7—C8—C12—C13'	−52.6 (9)
C11—C6—C7—C8	−0.9 (8)	C9—C8—C12—C13'	124.9 (8)

Symmetry code: (i) −*x*, −*y*+2, *z*.

### Hydrogen-bond geometry (Å, º)

**Table:**

*D*—H···*A*	*D*—H	H···*A*	*D*···*A*	*D*—H···*A*
O2—H2*B*···O2^ii^	0.82 (2)	2.07 (4)	2.821 (6)	153 (8)
O2—H2*A*···O3	0.81 (2)	2.26 (3)	2.806 (8)	125 (2)
O2—H2*A*···O3′	0.81 (2)	2.49 (4)	2.947 (10)	117 (3)
O2—H2*A*···N1	0.81 (2)	2.55 (3)	3.197 (6)	138 (3)

Symmetry code: (ii) *y*−1, −*x*+1, −*z*+1.
